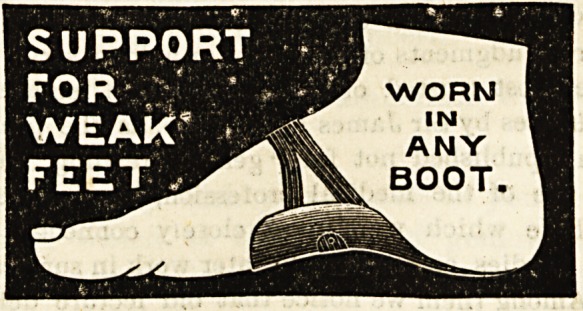# New Appliances and Things Medical

**Published:** 1902-06-14

**Authors:** 


### NEW APPLIANCES AND THINGS MEDICAL.

[We shall be glad to receive at our Office, 28 & 29 Southampton Street, Strand, London, W.C., from the manufacturers, specimens of all new preparations and appliances which may be brought out from time to time.]

### NEW CLAMP FOR CONTROLLING HÆMORRHAGE IN LIVER OPERATIONS.

(REYNOLDS AND BRANSON, LTD., 13 BRIGGATE, LEEDS.)

THIS new clamp is the invention of Dr. Stephens, of Rotherham. The instrument is 11 inches long, with 4½-inch blades. There is a strong racket catch and locked joint. The blades, which are made of two prongs, can be covered with rubber tubing, so as to protect the tissues from unnecessary damage, and at the same time prevent slipping. The use of this instrument will effectually control hæmorrhage when a wedge-shaped piece of liver is being removed, or in operations for gastrectomy, pylorectomy, or enterectomy, and at the same time it gives the operator or assistant the use of an additional hand, which would otherwise be required for the manual arrest of bleeding.

### PROTENE PREPARATIONS.

(PROTENE COMPANY, LIMITED, 36 WELBECK STREET, LONDON, W.)

We have more than once referred to the merits of the Protene Preparations in these columns. We would, however, on this occasion refer especially to the cocoa, which is the speciality of the firm. This cocoa is a well-devised mixture of high-class cocoa manufactured in this country, together with Protene, which is prepared from fresh milk and contains the most nutritious of its elements in a highly concentrated form. Protene Cocoa is a most delicious beverage, it is a highly concentrated food, and digestible by the most sensitive and delicate stomach. Its use is intended for invalids, children, and those who, for business or for pleasure, are exposed to serious muscular fatigue. The Protene travellers' lunch, which is another successful preparation introduced by this firm, is contained in a small cardboard box which slips easily into the pocket; it contains eight protene biscuits and two tablets of Protene chocolate, and represents the equivalent nutritive value of an ordinary full meal, such as is sufficient for an adult performing ordinary work. On the occasion of the late Queen's Diamond Jubilee these lunches were much appreciated by a large number of the spectators owing to the sustaining character of Protene and to the convenience with which this concentrated food can be carried in the pocket.

### THE IDEAL FOOT SUPPORT.

(J. POND, 21–23 CASTLE MEADOW, NORWICH.)

THE ideal foot support is a form of improved valgus pad to correct the tendency to flat foot which often occurs in persons of weakly constitution, and is always liable to present itself after middle age is past. The support fits comfortably under the arch of the instep, and is held in position by a strap which passes round the instep, and is fastened underneath by a special spring clasp. The ideal foot support can be worn with any boot, and by keeping up the arch of the instep prevents the distressing symptoms which accompany flat foot and weakness of the ligaments of the ankle. Those who have a tendency to these conditions, or who by reason of long hours of standing or walking experience a sense of weakness at the instep, will find much comfort in these improved valgus pads.

## Figures and Tables

**Figure f1:**
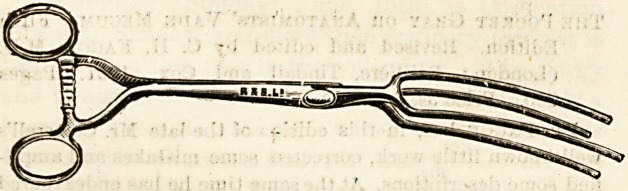


**Figure f2:**